# Big Pharma, Big Bucks, and a Big Pile o' Pigs

**DOI:** 10.1100/tsw.2001.9

**Published:** 2001-03-02

## Abstract

Nature leads this week with a story about the Bush administration's surprisingly liberal overtures to Third World countries that want to develop inexpensive AIDS medications. Efforts to unify the European research effort are the focus of Science's lead article.

AIDS has ravaged many African and South American countries in the past several years. But sufferers in developing countries cannot even afford Western retroviral AIDS cocktails at the lowest discounted prices, reports *Nature*. So health care workers in these countries have turned to other sources. For example, *Nature* reports that Father Angelo d'Agostino, head of Nyumbani Orphanage for children with HIV or AIDS in the Kenyan capital Nairobi, plans to start buying anti-AIDS drugs from the Indian company Cipla of Mumbai, which has promised to make the drugs available at about one-third of the minimum cost on offer from the Western companies. Although Big Pharma is not happy about the cheap drugs - 42 pharmaceutical companies are currently challenging a South African decision to allow domestic production of generic AIDS drugs - the previous US administration had adopted a "relaxed attitude" toward countries wishing to produce their own versions of expensive Western AIDS drugs, says *Nature*. The conservative new administration was expected to reverse this stance. But it now appears they will maintain the status quo. Speaking in *Nature*, Robert Zoellick, the US trade representative, promised to back efforts by the administration "to work with countries that develop serious programs to prevent and treat this horrible disease", provided such efforts were "consistent with our overall efforts to protect America's investment in intellectual property". The statement was predictably lauded by AIDS activists and downplayed by the Pharmaceutical Research and Manufacturers of America. *Science* did not cover this story.

Big numbers figure prominently in *Science*'s otherwise dry top story (and *Nature*'s similarly boring number four slot). And they are the numbers scientists like to hear: Four years and $16.2 billion. The European Commission approved the first draft of Framework 6 (FP6), a proposed 4-year $16.2 billion initiative designed to consolidate disparate national research programs, reports *Science*. The new proposal hopes to foster larger international collaborations and encourage scientists to move throughout the member countries. Chances are good that the European Parliament and the member countries will ratify the FP6 program within the year, says *Science*. If so, FP6 will replace the current Framework, FP5, in 2003. In contrast to the proposed FP6, FP5 supports large numbers of unrelated projects, reports *Nature*.

In other news, *Nature* and *Science* report that thousands of pigs had to be killed and roasted atop huge funeral pyres throughout the UK. The pigs were infected with foot-and-mouth disease, a generally non-fatal but extraordinarily infectious virus. It is the first major UK outbreak in 30 years, reports both journals. Even though the virus wouldn't have killed the animals, it reduces the amount of meat and milk produced by the pigs, so slaughtering the infected pigs before the virus spreads makes economic sense to farmers. Vaccines for foot-and-mouth exist, but they have one crucial drawback that makes farmers wary of using them. Occasionally they trigger another outburst.
